# Correction: Coming of age in recovery: The prevalence and correlates of substance use recovery status among adolescents and emerging adults

**DOI:** 10.1371/journal.pone.0318722

**Published:** 2025-01-30

**Authors:** Douglas C. Smith, Crystal A. Reinhart, Shahana Begum, Janaka Kosgolla, John F. Kelly, Brandon G. Bergman, Marni Basic

The sixth author’s middle initial is incorrect. The correct name is: Brandon G. Bergman. The correct citation is: Smith DC, Reinhart CA, Begum S, Kosgolla J, Kelly JF, Bergman BG, et al. (2023) Coming of age in recovery: The prevalence and correlates of substance use recovery status among adolescents and emerging adults. PLoS ONE 18(12): e0295330. https://doi.org/10.1371/journal.pone.0295330.

## References

[1] Smith DC, Reinhart CA, Begum S, Kosgolla J, Kelly JF, Bergman BB, et al. (2023) Coming of age in recovery: The prevalence and correlates of substance use recovery status among adolescents and emerging adults. PLoS ONE 18(12): e0295330. doi: 10.1371/journal.pone.0295330 38113212 PMC10729970

