# Supplementary material for: Coming of age in recovery: The prevalence and correlates of substance use recovery status among adolescents and emerging adults
Source: PLoS One. 2023 Dec 19;18(12):e0295330. doi: 10.1371/journal.pone.0295330 (PMC10729970; doi:10.1371/journal.pone.0295330)
Supplement: Attachment — Submitted filename: PlosOne_Response to Reviewers_Due July 17 2023.docx [file pone.0295330.s001.docx]

| **Author Responses to Reviewer Comments on Manuscript #PONE-D-23-08858**  **Manuscript Title:** “Coming of age in recovery: The prevalence and correlates of substance use recovery status among adolescents and emerging adults” | |
| --- | --- |
| **Reviewer Comments** | **Author Responses** |
| Reviewer #1: This is a well written paper on an important public health problem. The authors set out to describe the pattern of recovery among adolescents and youth in the US. The methods are well described and the findings well presented. The discussion is balanced and considers the study limitations.  I have one minor comment: The definitions/conceptualization of problem resolution and recovery did not come out well in the methods. The authors should provide a description of these terms to improve understandability. | We appreciate the kind comments on the paper’s novel contributions to the field.  We have tried to improve the clarity of the problem resolution and recovery terms by revising the section of the introduction entitled “Recovery Research with Adults.”  However, we also note that there is very limited work on defining these terms for adolescents. So, one of the main recommendations we have in the discussion section is to do more research on defining these terms. |
|  | |
| Reviewer #2: In the present study, the authors examined the prevalence of recovery and problem resolution in a sample of adolescents and young adults and the sociodemographic and behavioral characteristics associated with these. The study is well written and addresses an important yet understudied problem, however, I have several major concerns that would require further attention from the authors. | We appreciate the reviewer’s recognition that the study makes a novel contribution in an understudied area. We have worked hard to be as responsive to the methodological and conceptual comments made by Reviewer 2. |
| 1. The authors used the categorical variable recovery status as the outcome, however, I believe that the recovery status is supposed to be the exposure in this study. The hypothesis listed in introduction “given prior research, we hypothesized that youth in recovery would have more severe substance use and behavioral health problems relative to those not in recovery” supports the idea that the exposure is indeed recovery status and the outcome the sociodemographic and behavioral characteristics. This direction is further supported by claims made in the abstract “chi square, ANOVA, and regression analyses compared demographic variables, past 30 day and past year alcohol and other drug use, and behavioral outcomes among four groups of youth”, methods “use of any substance was used as the reference category for outcomes”, and results “3.3. Substance Use and Behavioral Health Outcomes in Relation to Recovery Identities”. |  |
| Moreover, the authors randomly selected a subset of individuals who responded no to both questions on recovery and problem resolution (NN group) but had used at least one substance in the past year, and used this subset of participants as the comparison group in their analyses. They proclaimed that “the random selection process successfully balanced demographics between the selected NN group and the three other recovery status groups”. The descriptive statistics in Table 1 indicate that this group differed from the other groups on most of the assessed characteristics. However, since recovery status was used as the outcome in the present version of the study, there should be a balanced covariate distribution between the exposed and unexposed group (e.g., individuals with 2+ CRAFFT score and individuals with lower CRAFT score). This also suggests that recovery status should be the exposure and sociodemographic and behavioral characteristics the outcome. | We have followed this reviewer’s suggestion to use propensity matching, which addresses this comment. We feel the resulting manuscript is much stronger. |
| While the reversal of exposure and outcome is generally discouraged since these should be, ideally, defined in the study protocol, I believe that the authors actually intended to use recovery status as the exposure and the sociodemographic and behavioral characteristics as the outcome. The authors should clarify what was the direction of the association that they intended to study. If indeed the outcome of interest were the sociodemographic and behavioral characteristics, then they should reverse the exposure-outcome pairs. | As noted above, we did this. Thank you for the suggestion. |
| 2. Regardless of what was the intended exposure and outcome, to increase the confidence that the observed effects are due to intrinsic differences between the exposed and unexposed group and not due to differences between these on covariates (ie, unaccounted confounding), the authors could utilize several methods and techniques that would make the exposed and unexposed group more comparable. Popular options that the authors could consider are propensity score matching (e.g., 10.1093/oxfordjournals.aje.a010011) or propensity score weighting (e.g., 10.1136/bmj.l5657). | We thank the reviewer for this excellent suggestion. Using optimal full matching, a form of propensity matching, we balanced covariates on (measured) potentially confounding variables. |
| 3. Putting aside whether these were the actual exposures, it seems to me that the authors included all exposures (i.e, sociodemographic and behavioral characteristics) in the regression models simultaneously, as indicated by Table 3 and 4. Such an approach would be problematic because it is likely that there are complex relationships between the included exposures: these could lead to spurious associations, including the reversal of the direction of the associations (e.g. 10.1097/EDE.0000000000001259, 10.1016/j.jclinepi.2022.05.021). | In this major revision, all confounds are accounted for in the match. Thus, the only variables in the models are the exposures (recovery groups) and outcomes (past 30 day substance use and binge drinking in the past two weeks.) |
| The authors should clarify whether they included all exposures in the models simultaneously. If so, then they should fit models containing the confounders and each of the exposure separately. | Optimal full matching addresses all the potential confounders in this analysis. |
| 4. I have no experiences with school-based surveys conducted in the United States, but it is a known limitation of general population surveys that people with substance use disorders, in particular those with severe ones, are substantially less likely to participate in them. | We appreciate this comment. We have added a sentence in the limitations section about this. |
| Is it possible that individuals who would have the most severe substance use disorders would not even be present at school, therefore would be unreachable by this survey? Complementarily, is it possible that these individuals would be less likely to participate in the survey when present in school? The authors should clarify these and expand the limitations section accordingly. | We appreciate this comment. We have added a sentence in the limitations section about this. |
| 5. The authors indicated that they used multiple imputation to impute the missing data, however, it is not clear what was present in the imputation model. Thus, the authors should elaborate what variables were used in the imputation model. | In this version of the manuscript, we clarified the imputation method (predictive mean matching in the R package, mice). This uses all variables in the dataset. |
| 6. I believe that the authors should provide more concrete steps on future development of the items on recovery from substance use in the population of adolescents and emerging adults. In particular, to ensure that each member of the target population has the same understanding of the underlying concept (i.e., recovery), the authors could provide potential directions for developing a definition of recovery in the context of adolescents and emerging adults. This would, then, ensure that the responses refer to the same concept and not multiple, potentially incomparable concepts. | We agree, and have proposed to use cognitive interviewing methods to check their understanding of these items. This would be a logical next step for item and scale development. |
