# Supplementary material for: Coming of age in recovery: The prevalence and correlates of substance use recovery status among adolescents and emerging adults
Source: PLoS One. 2023 Dec 19;18(12):e0295330. doi: 10.1371/journal.pone.0295330 (PMC10729970; doi:10.1371/journal.pone.0295330)
Supplement: Attachment — Submitted filename: Response to reviewers.doc [file pone.0295330.s002.doc]

School of Social Work

1010 W. Nevada Street

Urbana IL 61801

# University of Illinois

At Urbana-Champaign

Editor, Plos One

October 26^th^,2023

RE: Response to Reviewer Comments on PONE-D-23-08858R1

Dear Editor:

We were delighted that in your last correspondence you found merit in our paper entitled: **“Coming of age in recovery: The prevalence and correlates of substance use recovery status among adolescents and emerging adults (**PONE-D-23-08858R1).**”** Our understanding is that we need to respond to one outstanding reviewer comment.

Specifically, the reviewer raised concerns on our revised manuscript did not fully address their critique of our original manuscript. Specifically, they asked us to use propensity score matching to compare youth who resolved problems or were in recovery versus those who were not. We did this on our revised manuscript that we submitted in July 2023. However, concerns remained about our specific method of propensity score matching:

*“Reviewer #2: 1. to 3. Thank you for addressing my points. Using propensity score matching reduced the covariate imbalance between the groups, however, the authors performed matching also on past year substance use and CRAFT score. Since the outcome in models is 30-day substance use, by matching on covariates that are proxy for substance use, the authors de facto matched on the outcome. This can be responsible for detecting mostly null effects in the regression models. Thus, the authors should rerun the matching without matching on substance use and CRAFT score.”*

In response to this comment, we reran models following this suggestion, and had a discussion with all co-authors. Although the reviewer is right that some statistical dependency may exist when including past year substance use in the match, this method answers a more interesting research question. For this and other reasons listed below, we feel strongly that our R1 analysis was appropriate and should be used as the published result. Our two main reasons include:

1. The R1 analysis answers the question: Relative to youth with a similarly high level of substance use and substance use consequences in the past year, do youth who say they are in recovery or have resolved problems have lower odds of pas month substance use? The co-authors agree that any significant differences showing one of the recovery/problem resolution groups having lower odds of past month use, could be construed as validity data for the concept of recovery/problem resolution, which requires some degree of remission. This is because they are more likely to be abstinent in the past month relative to matched controls with similar substance use.

For theoretical reasons, we feel this is much more interesting research question that what the reviewer proposes we do by changing matching procedures. If we followed the reviewer comment and eliminated past year substance use and consequences in the match the research question changes. It becomes: Relative to youth who have lower levels of substance use, but are matched on demographics, do youth resolving problems or reporting being in recovery have lower odds of past month substance use?

Because youth in recovery are thought to have had serious substance use and then a period of remission, it makes more sense to compare them to a matched group with similar use. In other words, for youth that are in recovery more recent use implies a relapse on substances, but if you compare them to youth without serious substance use, you are just comparing the presence or absence of use, which has a very different meaning for youth without serious substance use issues. To illustrate this, we attached findings from the analysis that follows the reviewer suggestion in question. As you can see the first column of table 2 below that shows that the general population (“Sample”) has much lower past year use than the recovery and problem resolving groups. Thus, we maintain that the best comparator here would consist of youth with similarly high substance use, as achieved in the matching we did in the R1 submission.

2. Contrary to what the reviewer hypothesized, when we did the match after excluding past year substance use variables and the CRAFFT measure, we had entirely null findings (See logistic regression results at bottom of this letter). The reviewer suggested that our R1 methods were perhaps inhibiting finding more statistically significant differences. That is not the case. This contradicts the idea that null findings are entirely from statistical dependencies. *Nevertheless, because of this comment, we added a note about this as a limitation on page 18.*

With all due respect, we feel that the new analyses we completed based on this comment show that the logic of the comment was incorrect. Although we cannot know if additional comparisons would have been statistically significant with a better methodology, we feel the most appropriate course is to retain the R1 analysis and acknowledge this as a limitation that can be addressed in future work.

Sincerely,

Douglas C. Smith, Ph.D.

Professor, School of Social Work

University of Illinois at Urbana Champaign

1010 W. Nevada, MC-082

Urbana, IL 61801

217-333-4185

[smithdc@illinois.edu](mailto:smithdc@illinois.edu)

**Analyses Below Respond to Reviewer Comment to remove substance use and CRAFFT from propensity match (DO NOT USE IN PUBLISHED VERSION)**

**Figures**

a)

b)

c)

**Figure 1:** *Covariate balance for the treatment group a) recovery only (RO), b) problem resolution (PRO), c) dual status (DS)*

a)

b)

c)

**Figure 2:** *The logit coefficient estimate of the Average Treatment Effect on the Treated (ATT) on the matched sample for the treatment group a) recovery only (RO), b) problem resolution (PRO), c) dual status (DS)*

*Note:	*** p < 0.05: Significant*

Table 1. Student characteristics in the sample and among the recovery groups

|  | **Sample** | **Dual Status (DS)** | **Recovery Only (RO)** | **Problem Resolution (PRO)** |  |
| --- | --- | --- | --- | --- | --- |
|  |  |  |  |  |  |
|  | **N (%)** | **N (%)** | **N (%)** | **N (%)** |  |
| **All** | 56650 | 884 | 1811 | 1546 |  |
| **Gender** |  |  |  |  |  |
| Female | 28853 ( 50.9) | 399 | 864 | 685 (44.3) |  |
| Male | 26820 (47.3) | 420 | 871 | 768 (49.7) |  |
| Transgender | 402 (0.7) | 31 | 33 | 43 (2.8) |  |
| Do not identify | 575 (1.0) | 34 | 43 | 50 (3.2) |  |
| **Race** |  |  |  |  |  |
| White | 38233 (67.5) | 552 (62.4) | 818 (45.1) | 1067 (69.0) |  |
| Black/African American | 3121 (5.5) | 54 (6.1) | 158 (8.7) | 101 (6.5) |  |
| Latino/Latina | 8555 (15.1) | 162 (18.3) | 605 (33.4) | 228 (14.7) |  |
| Asian American | 3722 (6.5) | 39 (4.4) | 92 (5.1) | 43 (2.8) |  |
| Any Other | 3019 (5.3) | 77 (8.7) | 138 (7.6) | 107 (6.9) |  |
| **Age (N, mean)** | 56650 ( 16.1) | 884 (16.3) | 1811 (16.1) | 1546 (16.3) |  |
| **Grade** |  |  |  |  |  |
| 9^th^ | 8849 (14.9) | 86 (9.7) | 283 (15.6) | 166 (10.7) |  |
| 10^th^ | 23289 (41.1) | 357 (40.4) | 795 (43.9) | 586 (37.9) |  |
| 11^th^ | 7735 (13.6) | 139 (15.7) | 252 (13.9) | 223 (14.4) |  |
| 12^th^ | 17177 (30.3) | 302 (34.2) | 481 (26.5) | 571 (36.9) |  |
| **Free/reduced lunch** |  |  |  |  |  |
| Free lunch | 15103 (26.7) | 340 (38.4) | 847 (46.8) | 516 (33.3) |  |
| Reduced price lunch | 3836 (6.8) | 69 (7.8) | 178 (9.8) | 106 (6.8) |  |
| Neither | 37711 (66.5) | 475 (53.8) | 786 (43.4) | 924 (59.7) |  |
| **Region** |  |  |  |  |  |
| Suburban Chicago | 40507 (71.5) | 589 (66.6) | 1269 (70.1) | 1018 (65.8) |  |
| Chicago* | 871 (1.5) | 25 (2.8) | 68 (3.7) | 19 (1.2) |  |
| Other Urban | 8607 (15.1) | 158 (17.9) | 263 (14.5) | 291 (18.8) |  |
| Rural | 6642 (11.7) | 112 (12.7) | 211 (11.7) | 218 (14.1) |  |

**Chicago data in 2020 was very low due to COVID*

Table 2. Substance use, other high-risk behaviors, and mental health across the recovery groups

|  | **Sample** | **Dual Status** | **Recovery Only** | **Problem Resolution Only (PRO)** |
| --- | --- | --- | --- | --- |
|  |  | **(DS)** | **(RO)** |  |
|  | **N (%)** | **N (%)** | **N (%)** | **N (%)** |
| **All** | 56650 | 884 | 1811 | 1546 |
| **Substance use in the past 30 days** | | |  |  |
| Alcohol | 824 (1.5) | 125 (14.1) | 102 (5.6) | 166 (10.7) |
| Marijuana | 2555 (4.5) | 246 (27.8) | 210 (11.6) | 393 (25.4) |
| Rx drugs not prescribed | 1172 (2.1) | 221 (25.0) | 151 (8.3) | 282 (18.2) |
| **Binge drinking over the past two weeks** | 591 (1.0) | 103 (11.6) | 89 (4.9) | 157 (10.1) |
| **Substance use in the past year** | |  |  |  |
| Alcohol | 5204 (9.2) | 325 (36.8) | 246 (14.5) | 367 (23.7) |
| Marijuana | 4741 (8.4) | 404 (45.7) | 323 (17.8) | 703 (45.4) |
| Inhalants | 407 (0.7) | 102 (11.5) | 62 (3.4) | 125 (8.1) |
| MDMA, Ecstasy | 307 (0.5) | 92 (10.4) | 62 (3.4) | 130 (8.4) |
| LSD | 349 (0.6) | 96 (10.9) | 66 (3.0) | 146 (9.4) |
| Cocaine | 342 (0.6) | 100 (11.3) | 67 (3.7) | 128 (8.2) |
| Methamphetamines | 297 (0.5) | 95 (10.7) | 59 (3.2) | 121 (7.8) |
| Heroin | 319 (0.5) | 89 (10.0) | 58 (3.2) | 113 (7.3) |
| Rx drugs not prescribed | 1891(3.3) | 305 (34.5) | 187 (10.3) | 399 (25.8) |
| Over the counter drugs | 407 (0.5) | 141 (15.9) | 80 (4.4) | 176 (11.4) |
| Rx painkillers to get high | 362 (0.6) | 144 (16.3) | 77 (4.2) | 195 (12.6) |
| Other Rx drugs to get high | 665 (11.9) | 216 (24.4) | 101 (5.6) | 267 (17.3) |
| **Mental Health and CRAFFT** | |  |  |  |
| Depressive symptoms | 18581 (32.8) | 219 (24.8) | 974 (53.7) | 964 (61.1) |
| Suicidal ideation | 7772 (13.6) | 381 (43.0) | 515 (28.4) | 589 (38.0) |
| 2+ CRAFFT score | 9378 (16.5) | 141 (15.9) | 768 (42.4) | 413 (26.7) |
| **Gambling** |  |  |  |  |
| Gambled via machine | 1518 (2.7) | 165 (18.7) | 147 (8.1) | 213 (13.8) |
| Gambled via online | 2148 (3.8) | 187 (21.2) | 179 (9.9) | 240 (15.5) |
| Felt bad about gambling | 2135 (3.8) | 208 (23.5) | 220 (12.1) | 223 (14.4) |
| Gamble more than planned | 1655 (2.9) | 217 (24.5) | 160 (8.8) | 226 (14.6) |
| **Conduct Problems** |  |  |  |  |
| Physical fight | 8052 (14.2) | 183 (20.7) | 548 (30.2) | 621 (40.1) |
| Carried weapon | 4711 (8.3) | 195 (22.0) | 320 (17.7) | 487 (31.5) |
| Sold illegal drugs | 1713 (3.0) | 197 (22.2) | 228 (12.5) | 449 (29.0) |
| Been drunk or high | 4224 (7.4) | 365 (41.3) | 472 (26.1) | 738 (47.7) |

**References**

Abadie, A., & Imbens, G. W. (2006). Large Sample Properties of Matching Estimators for Average Treatment Effects. Econometrica, 74(1), 235-267.

Austin, P. C. (2011). An Introduction to Propensity Score Methods for Reducing the Effects of Confounding in Observational Studies. Multivariate Behavioral Research, 46(3), 399-424.

Hansen, B. B., & Klopfer, S. O. (2006). Optimal Full Matching and Related Designs via Network Flows. Journal of Computational and Graphical Statistics, 15(3), 609–627. doi:10.1198/ 106186006X137047.

Ho, D. E., Imai, K., King, G., & Stuart, E. A. (2011). MatchIt: Nonparametric Preprocessing for Parametric Causal Inference. Journal of Statistical Software, 42(8), 1-28.

Rosenbaum, P. R., & Rubin, D. B. (1983). The central role of the propensity score in observational studies for causal effects. Biometrika, 70(1), 41-55.

Stef van Buuren, Karin Groothuis-Oudshoorn (2011). mice: Multivariate Imputation by Chained Equations in R. Journal of Statistical Software, 45(3), 1-67. DOI 10.18637/jss.v045.i03.
